# Vanishing testes syndrome-related osteoporosis and high cardio-metabolic risk in an adult male with long term untreated hypergonadotropic hypogonadism

**DOI:** 10.1590/2359-3997000000127

**Published:** 2016-01-01

**Authors:** Mara Carsote, Cristina Capatina, Ana Valea, Anda Dumitrascu

**Affiliations:** 1 C.I. Parhon National Institute of Endocrinology Bucharest Romania C.I. Parhon National Institute of Endocrinology, Bucharest, Romania; Carol Davila University of Medicine and Pharmacy Bucharest Romania & Carol Davila University of Medicine and Pharmacy, Bucharest, Romania; 2 Iuliu Hatieganu University of Medicine and Pharmacy Iuliu Hatieganu University of Medicine and Pharmacy; Clinical County Hospital Cluj-Napoca Romania & Clinical County Hospital, Cluj-Napoca, Romania; 3 C.I. Parhon National Institute of Endocrinology Bucharest Romania C.I. Parhon National Institute of Endocrinology, Bucharest, Romania

## Abstract

The male hypogonadism-related bone mass loss is often under diagnosed. Peak bone mass is severely affected if the hypogonadism occurs during puberty and is left untreated. We present an interesting; almost bizarre case of a male with non-functional testes early during childhood and undiagnosed and untreated hypogonadism until his fifth decade of life. Forty six year male is referred for goitre, complaining of back pain. Phenotype suggested intersexuality: gynoid proportions, micropenis, no palpable testes into the scrotum, no facial or truncal hair. His medical history had been unremarkable until the previous year when primary hypothyroidism was diagnosed and levothyroxine replacement was initiated. Later, he was diagnosed with ischemic heart disease, with inaugural unstable angina. On admission, the testosterone was 0.2 ng/mL (normal: 1.7-7.8 ng/mL), FSH markedly increased (56 mUI/mL), with normal adrenal axis, and TSH (under thyroxine replacement). High bone turnover markers, and blood cholesterol, and impaired glucose tolerance were diagnosed. The testes were not present in the scrotum. Abdominal computed tomography suggested bilateral masses of 1.6 cm diameter within the abdominal fat that were removed but no gonadal tissue was confirmed histopathologically. Vanishing testes syndrome was confirmed. The central DXA showed lumbar bone mineral density of 0.905 g/cm^2^, Z-score of -2.9SD. The spine profile X-Ray revealed multiple thoracic vertebral fractures. Alendronate therapy together with vitamin D and calcium supplements and trans-dermal testosterone were started. Four decades of hypogonadism associate increased cardiac risk, as well as decreased bone mass and high fracture risk.

## INTRODUCTION

Male osteoporosis represents a public health problem. The clinical risk factors and the diagnosis itself are often under-recognised (1,2). In one half of cases the aetiology is identified: glucocorticoid treatment, heavy smoking or alcohol consumption, medical conditions such as chronic inflammatory diseases, etc. (1,2). Hypogonadism represents 5% up to 15% of all causes and three patterns are described: iatrogenic hypogonadism for prostate cancer control, progressive adult-onset age-related hypogonadism, typically with mild testosterone deficiency, severe hypogonadism in genetic or endocrine conditions (with either low or high serum gonadotropins), usually diagnosed in young patients (1,3). The hypogonadism with very early onset which persists into adulthood is associated not only with a decreased pubertal peak bone mass but also with a high bone turnover state and an increased risk of falling (due to the decreased muscle strength and possible associated vitamin D deficiency), all leading to fragility fractures. In adult men low testosterone levels are an independent risk factor for hip fracture especially in the elderly, and the replacement therapy has a beneficial effect, increasing the lumbar and hip bone mineral density (BMD) (4,5). The hypogonadism is a common pathway between the bone and metabolic complications and impaired quality of life.

## CASE REPORT

A 46-years non-smoker Caucasian male, originating from an iodine-deficiency endemic area, previously treated for one year for primary hypothyroidism was referred for further goitre evaluation. He was the youngest of five siblings (four brothers and one sister), none with remarkable medical records.

### Case history

At the time of the initial diagnosis of primary hypothyroidism, his serum concentration of thyroid stimulating hormone (TSH) was markedly elevated (116 micro international units per millilitre, µUI/mL; reference range 0.46-4.68 µIU/mL). Daily levothyroxine was initiated in progressive doses up to current dose of 100 micrograms (µg). On admission his only complaints were intermittent back pain for the last 2-3 years and rare episodes of mild mastalgia since adolescence, for which he received no therapy. The medical history revealed stage III arterial hypertension (diagnosed 2 years before, partially controlled under angiotensin converting enzyme inhibitor, calcium blocker and diuretic treatment). Ischemic chronic heart disease was also present: the patient suffered one year before admission (two months after levothyroxine was started) an episode of severe unstable angina which led to his forced early retirement (was previously employed as a construction worker). He was started on a daily regimen of 75 miligrams (mg) of clopidogrel, orally nitrates, and atorvastatin for high blood cholesterol.

### Clinical examination

The clinical exam found intersexuality features: gynoid fat distribution with waist girth of 94 centimetres (cm), hip girth of 109 cm, waist to hip ratio of 0.86, infantile penis, no palpable testes into the scrotum or inguinal canal, bilateral gynecomastia, present axillary and pubic hair, but no facial or truncal hair. His height was 164 cm and he was mildly obese (body mass index 31.23 kilograms/square meters -kg/m^2^). He had a normal intelligence quotient, and he denied ever having any sexual activity, as well as ever experiencing sexual attraction to either sex. He had a mild progressive kyphosis and his gonadal or bone metabolism status had never been investigated.

### Paraclinical assessment

**Figure 1:**
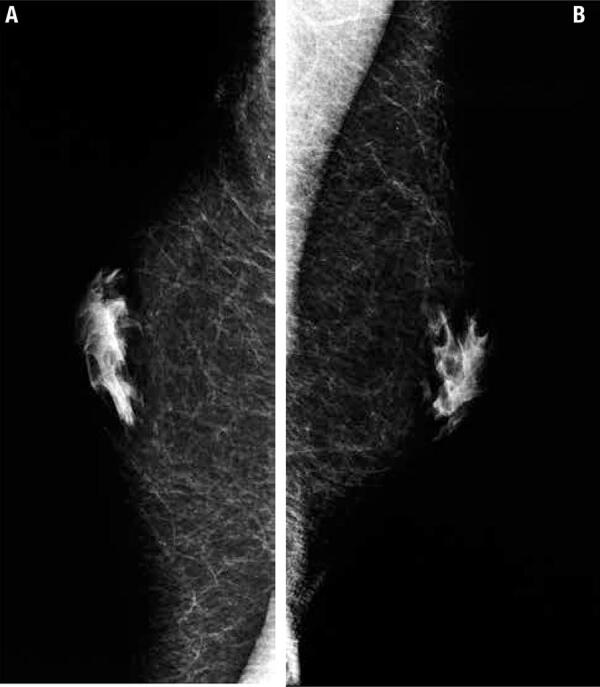
Mamography: bilateral gynecomastia in a 46 year male with hypogonadism. (A) Left gynecomastia. (B) Right gynecomastia.

The cardio-metabolic profile revealed low normal high density lipoprotein (HDL) cholesterol, impaired glucose tolerance high uric acid levels (6). The ophthalmological examination diagnosed stage II retinal angiosclerosis. The total testosterone level (measured by chemiluminescence) was extremely low (0.2 ng/mL) with high follicle stimulating hormone (FSH). Mild anaemia was correlated to hypogonadism. Normal adrenal axis as well as TSH level (under levothyroxine substitution) was found (Table 1). The peripheral blood karyotype was tested twice and found with the same result: 46, XY. The breast ultrasound and the mammography confirmed bilateral gynecomastia (Figure 1).

**Table 1:** The biochemistry and endocrine parameters in a 46-old male with long term (more than 40 years) undiagnosed and untreated hypogonadism

Parameter	Measured concentration	Normal values	Units

Biochemistry assays			
Total cholesterol*	127		Milligrams/decilitre (mg/mL)
HDL-cholesterol	38	35-55	mg/dL
Fasting plasma glucose	124	70-110	mg/dL
2-hour plasma glucose**	157	< 140	mg/dL
Glycated Haemoglobin A1c	6	4.8-5.7	%
Uric acid	7.14	3.5-7	mg/dL
Haemoglobin***	12.1	14-17%	Grams/decilitre (g/dL)
Hematocrit	35.8	41-53	%

**Hormonal panel **			

Total testosterone	0.21	1.75-7.81	Nanogram/millilitre (ng/mL)
Plasma estradiol	26	< 20	Picograms/millilitre (pg/mL)
FSH (follicle stimulating hormone)	56	1.27-19.26	mUI/mL
LH (luteinizing)	6	1.24-8.62	mIU/mL
Androstenedione	1.51	0.45-4.2	ng/mL
DHEA (dehydroepiandrosteron)	8.29	1.8-12.5	ng/mL
Prolactin	15	2.64-13.13	Nanogram/millilitre (ng/mL)
TSH (thyroid stimulating hormone)****	4.2	0.5-4.5	Micro international units per millilitre (µIU/mL)
Free T4 (thyroxine)	16	10.3-24.4	Picomol/litre (pmol/L)
Serum thyroperoxidase (TPO) Antibodies	64	0-35	IU/mL
Morning plasma ACTH (adrenocorticotrophic hormone)	27	3-66	pg/mL
AMH (Anti-Mullerian hormone)	0.08	1.3-14.8	ng/mL
SHBG (Sex hormone binding globulin)	27.91	14.5-48.4	Nanomol/litre (nmol/L)

**Neuroendocrine markers**			

Serum calcitonin	1	1-11.8	pg/mL
PSA (specific prostatic antigen)	0.02	0-4	ng/mL
Neuronal specific enolase	11.8	0-12	ng/mL
Beta-HCG (human chorionic gonadotropin)	0.06	0.5-2.67	mIU/mL

**Bone metabolism**			

Total serum calcium	9.8	8.5-10.2	mg/dL
Total serum phosphorus	3.69	2.5-4.5	mg/dL
PTH (parathormone)	24	15-65	pg/mL
25-hydroxyvitamin D	19	30-100	ng/mL
Serum osteocalcin	1.02	0.142-0.584	ng/mL
Serum CrossLaps	14.98	14-42	ng/mL

* Under daily 20 mg of atorvastatin; ** 75-grams oral glucose tolerance test (OGTT); *** Normocytic normochromic anaemia; **** Under daily 100 micrograms (µg) of levothyroxine.

**Figure 2:**
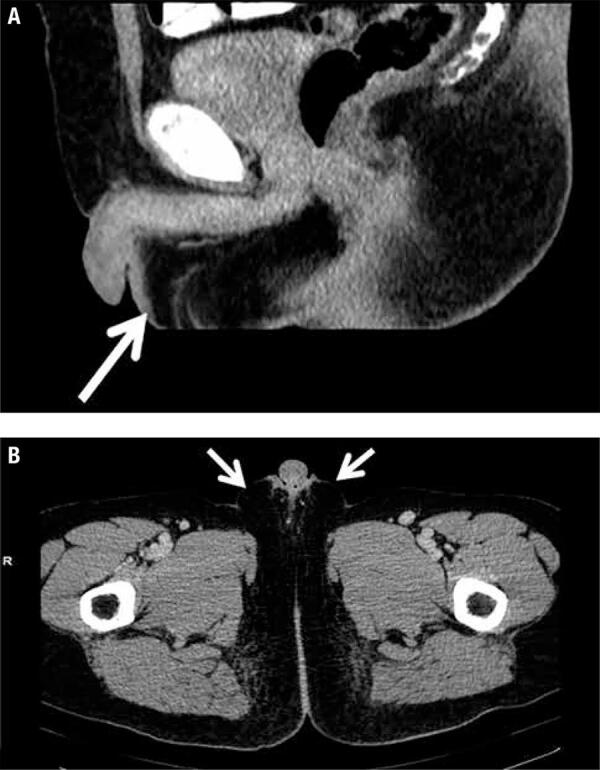
Computed tomography: bilateral absence of testes within the scrotum (arrow). (A) Sagittal plan. (B) Transversal plan.

The testes ultrasound did not show any gonads within the scrotum or the inguinal canal and neither did the abdominal and pelvic computed tomography (CT) (Figure 2). CT examination suggested bilateral nodes of maximum 1.6 cm into the abdominal fat that were later laproscopicaly removed but the pathological exam only described nonspecific reactive lymph nodes and no gonadal tissue. The karyotype was 46, XY. Based on all these data, the diagnosis of vanishing testes syndrome was made.

**Figure 3:**
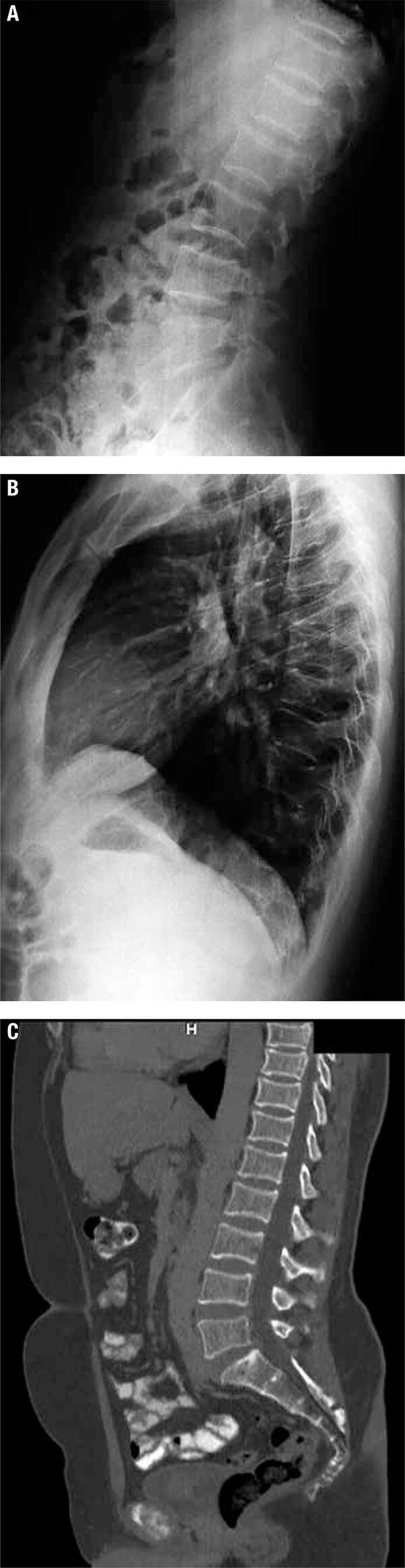
Vertebral fractures (A+B+C). (A) Profile lumbar X-Ray. (B) Profile thoracic X-Ray. (C) Computed tomogaphy image of the spine.

The bone assessment was compatible with severe secondary osteoporosis. Normal calcium and phosphorus levels, with elevated serum bone turnover markers (osteocalcin as bone formation marker and CrossLaps as bone resorption marker) (Table 1). The spine profile X-Ray revealed multiple vertebral fractures, predominantly in the thoracic spine with reduced vertebral height, confirmed at CT (Figure 3). The central DXA (GE Lunar Prodigy machine) showed decreased lumbar L2-4 BMD with Z-score of -2.9 SD. Total hip BMD was 1.1105 g/cm^2^, Z-score of 0.2SD; the femoral neck BMD was 0.996 g/cm^2^, Z-score of -0.2SD. The grip strength using a portable handheld dynamometer Kern MAP80K1 was very low for a man (comparable to the expected levels of a woman by the same age): of 29.5 at right hand with normal of 48 (40-57) for men, and 30 (25.8-35) for women; and of 27.7 at left hand with normal of 50 (42-58) for men, respective 34 (29-39) for women) (7). The quality of life was evaluated with the EuroQol questionnaire.

### Management

Secondary severe osteoporosis was diagnosed and therapy with alendronate 70 mg weekly, daily vitamin D and calcium supplements as well as non scrotal testosterone gel (5 mg daily) was initiated. The patient also continued the cardiovascular medication. Close follow-up was advised.

## DISCUSSION

This is a most unusual case of male hypogonadism undiagnosed and untreated for more than 40 years. On admission the most important practical problems were to identify the etiopathogenic form of hypogonadism; to search for potential gonadal dysgenesis-related tumours; to diagnose and to treat the bone and metabolic complications associated with severe long term testosterone deficiency.

The initial challenge in approaching the patient was the cause of the testicular dysfunction. The 46, XY karyotype, the normal male phenotype at birth, the failure to later develop male secondary sex characteristics (with infantile penis measuring 6 cm in the adult age) represent arguments for a very early childhood onset hypogonadism. The normal male appearance at birth with no intersexuality features and no Müllerian ducts derivatives prove that functional AMH (Anti-Müllerian hormone) activity had been present during foetal life (8) and make** c**ongenital bilateral anorchia unlikely. The almost undetectable value of AMH in a phenotypic male with 46 XY karyotype, bilateral cryptorchidism and no Müllerian ducts derivates is suggestive of late prenatal or early postnatal anorchia and not bilateral testes ectopia. We had no records of patient’s testicular trauma after birth or his development during puberty, neither any medical data related to his mother during pregnancy. The presented case seems to be an extreme phenotype of male 46, XY hypogonadism with a very early onset, left undiagnosed for 4 decades.

Although CT suggested some possible gonad tissue within the abdominal fat, after the surgical procedure the pathologic report did not confirm it. Since no ectopic testes tissue was found, according to the clinical, ultrasound and CT examination the diagnosis of vanishing testes syndrome was confirmed at most unusual age of 46. The disease, also named testicular regression syndrome is of unknown cause and the presumed pathogenesis relates to late antenatal or perinatal testicular insult, resulting in the shrinkage of the testes early in life (9). Some alternative imaging procedures have been suggested to differentiate the ectopic from in situ dysgenetic gonads with different blood vessels distribution and to avoid unnecessary surgery: testes venography, pelvic magnetic resonance imagery combined with arteriography, but these are more useful at a much younger age (10,11). In the last decades the technical advances of the testes ultrasound greatly limited their use (12). Yet, some authors considered that exploratory laparotomy is more useful than imaging methods for finding possible gonad remnants or associated tumours in impalpable testes (13). Based on the results from ultrasonography, CT and the exploratory laparoscopy we were not able to find any gonadal remnants. The endocrine tests suggested absent testicular function (low AMH, low to undetectable testosterone, high FSH and luteinizing hormone) so acquired bilateral anorchia was considered (14). In rare cases with persistent gonadal remnants, germ cells have been found in testicular remnants, with a theoretical impact on both assisted fertility and germ-cell neoplasia potential (9,15,16). However, fertility is highly unlikely after such a long time interval since disease onset; even more, others argue that the testicular biopsy as well as the human chorionic gonadotrophin stimulation test have limited therapeutic utility (15,16). In the case of regression testes syndrome most of the authors agree that the testicular remnants (if any) are not associated with germ line neoplasia (9). Since the neuroendocrine markers (Neuronal specific enolase, Beta-HCG) and CT excluded an abdominal or pelvic tumour, no secondary urological procedure was indicated in our case.

Our patient had been previously diagnosed with autoimmune primary hypothyroidism which is an atypical finding in empty scrotum syndrome; most probably this is a coincidental occurrence and there is no pathogenic correlation. The cardio-metabolic high risk profile is justified by the high blood pressure, chronic ischemic heart disease with a history of unstable acute angina, impaired glucose tolerance, obesity, dyslipidemia, increased uric acid concentration due to the persistent testosterone deficiency. Classical forms of late onset male hypogonadism are associated with a higher mortality because of the impact of the low testosterone status on glucose metabolism and cardiovascular system, expressed as insulin resistance, type 2 diabetes mellitus, hyperlipidemia or high blood pressure (17,18). There is an inverse relationship between testosterone levels and obesity or weight circumference. The production of estradiol from testosterone by aromatization in adipose tissue further suppresses the testosterone production via a central hypothalamic-pituitary-testes axis mechanism (19). In cases such as ours with severe hypergonadotropic hypogonadism the only source of androgens are the adrenals. The normal weak androgens secreted by the adrenals [dehydroepiandrosteron (DHEA) and especially androstenedione] are converted by aromatase into estrogens which exert both positive effects (on bone) and negative ones (gynecomastia). Our patient had normal adrenal androgens and increased estradiol. Due to the advanced age at diagnosis, the full-blown picture of the metabolic syndrome was present. In adult male hypogonadism sexual dysfunction is associated. Interestingly in this case the patient had no sexual activity, preferences, sexual interest or needs probably due to the effects of early testosterone deficiency on brain development (20).

The severe male osteoporosis is a common complication of long term untreated hypogonadism. Androgens exert their protective effects, increasing or preserving the BMD at all stages of human development. Both early and late hypogonadism influence the BMD, either impairing the acquisition of a normal pubertal peak bone mass or increasing the rate of bone loss (21). In our case we found multiple vertebral fractures and a high bone turnover status, low lumbar BMD but total hip and femoral neck BMD were unaffected. The increased risk of falling in early-onset hypogonadism is related to low testosterone levels, the vitamin D deficiency, and both of them induce reduced muscle strength. Potential variations of blood pressure and glycaemia levels may contribute to fall in this particular case.

In experimental models a direct androgenic effect on trabecular bone, independent of the stimulation of the estrogen receptors on both osteoblasts and osteocytes was described (22). The androgens also exert a strong antifracture protection by extraskeletal mechanisms related to muscle mass and strength (22). We checked the grip strength of our patient (by hand-held dynamometer) and recorded abnormally low levels for the male sex, within the normal ranges for a woman of the same age. The 25-hydroxyvitamin D of 19 ng/mL confirmed the concurrent vitamin D deficiency (probably related to the low sun exposure in this particular case), consistent with the association between metabolic syndrome and the testosterone deficiency syndrome (23).

The bone status as well as metabolic complications is expected to improve after the initiation of both testosterone replacement (24) and antiresorbtive therapy. For both osteoporosis and hypogonadism management the patient preferred non-injectable products.

## CONCLUSIONS

This case, characterised by the most unusual diagnosis at age of 46 of a the rare early-onset vanishing testes syndrome, demonstrates the large panel of complications developed in a lifetime of untreated testosterone deficiency from no sexual orientation or interest to bone loss and cardio-metabolic high risk profile.
